# *Rhododendron* Microshoot Culture as a Source of Phenolic Antioxidants for Biomedicine

**DOI:** 10.3390/molecules30142949

**Published:** 2025-07-12

**Authors:** Vera M. Katanskaya, Olga G. Vasilyeva, Elena P. Khramova, Natalia N. Sazhina, Evgenia A. Goncharuk, Tatiana L. Nechaeva, Maria Y. Zubova, Maria A. Aksenova, Petr V. Lapshin, Natalia V. Zagoskina

**Affiliations:** 1 K.A. Timiryazev Institute of Plant Physiology of the Russian Academy of Sciences, 127276 Moscow, Russia; goncharuk.ewgenia@yandex.ru (E.A.G.); nechaevatatyana.07@yandex.ru (T.L.N.); mariia.zubova@yandex.ru (M.Y.Z.); aksenova@ifr.moscow (M.A.A.); p.lapshin@mail.ru (P.V.L.); 2 N.V. Tsitsin Main Botanical Garden of the Russian Academy of Sciences, 127276 Moscow, Russia; olgozerova@yandex.ru; 3 Central Siberian Botanical Garden of the Siberian Branch of the Russian Academy of Sciences, 630116 Novosibirsk, Russia; elenakhramova2023@yandex.ru; 4 N.M. Emanuel Institute of Biochemical Physics of the Russian Academy of Sciences, 119334 Moscow, Russia; natnik48s@yandex.ru

**Keywords:** *Rhododendron* L., micropropagation, polyphenols composition, antioxidant activity

## Abstract

The search for alternative sources of biologically active compounds of plant origin, including phenolic compounds (PCs), is of great importance in medicine and pharmacology. Morphophysiological characteristics, photosynthetic pigments, PCs content, phenolic profile, as well as antioxidant (AOA) and antiradical activity (ARA), were studied for in vitro rhododendrons’ microshoots (*R. smirnowii*, *R. PJM Elite*, *R. japonicum*). The microshoots of *R. PJM Elite* had the highest photosynthetic pigments content (chlorophylls *a* and *b*), exceeding that of *R. smirnowii* and *R. japonicum*, it was 33% and 42%, respectively. The total phenolic content increased in the row *R. PJM Elite* < *R. smirnowii* < *R. japonicum.* Twelve to twenty phenolic compounds were identified in ethanol extracts of rhododendron microshoots, using high-performance liquid chromatography. Quercetin, kaempferol, and myricetin dominated in the phenolic complex of *R. japonicum* and *R. smirnowii*, whereas in *R. PJM Elite*, they were taxifolin and (−)-epicatechin. The AOA and ARA evaluation in DPPH-radical system and the model of initiated liposomes oxidation allowed to determine the highest activity in both systems for R. *japonicum* extracts, which was not typical for the other two species extracts. A high correlation was found between AOA extracts and the flavonoid content in them. The results obtained indicate the prospects of using *R. japonicum* and *R. PJM Elite* microshoots as an alternative source of flavonols and flavanols, accordingly.

## 1. Introduction

One of the characteristic features of higher plants is the ability to form phenolic compounds (PCs), one of the most common secondary metabolites [1,2]. According to modern screening data, more than 10,000 of their representatives have been identified, differing in structure, chemical properties, and biological activity [3,4].

PCs are involved in the processes of photosynthesis and respiration, phototropism, protection of the photosynthetic and genetic apparatus from UV-B radiation, pathogens, and phytophagouses, as well as adaptation to various stressful influences [5,6,7,8].

These secondary metabolites represent highly effective low-molecular-weight (non-enzyme) antioxidants that protect cells from the effects of free radicals (O_2_^•−^, NO_2_^•^, HO^•^), hydrogen peroxide (H_2_O_2_), and singlet oxygen (1O_2_), which initiate oxidative stress [9,10,11]. PCs inhibit non-specific redox reactions by directly interacting not only with radicals, but also with intermediates of phospholipid and fatty acid oxidation [12,13]. The most effective antioxidants of phenolic nature are flavonoids [14,15]. In recent years, there has been increasing evidence on the antioxidant potential of phenylpropanoids [16,17].

PCs are widely used for human health protection, namely in the prevention and treatment of diseases caused by the action of various stress factors and leading to the development of oxidative stress in the membranes of blood cells, liver, brain, and other organs [18,19]. The cardio- and hepatoprotective role of PCs [20,21], their immunomodulatory and antiviral effects, including against SARS-CoV-2, have been reported [22,23].

Valuable medicinal plants include genus *Rhododendron* L., which is one of the largest taxons of the Ericaceae DC, numbering more than 1200 species represented by evergreen, semi-deciduous, and deciduous shrubs, rarely trees [24,25]. Rhododendrons are widespread in China, Japan, India, and North America. In Europe, they grow mainly in the Alps, Pyrenees, and Scandinavia, and in Russia—in the Caucasus, Siberia, and the Far East [25,26,27]. Rhododendrons occupy high-altitude and coastal areas with temperate and cold climates, acidic, aerated soils, and high air humidity. Due to the high adaptive potential characteristic of these plants, they have been successfully introduced in many countries across the world [28,29].

Researchers’ interest in plants of the genus *Rhododendron* L. is due to their decorative properties, resistance to various biotic and abiotic stressors, and phytochemical composition [29,30,31]. They are characterized by a high ability to form PCs, depending on the species of plants, the stage of their ontogenetic development, and organospecificity [32,33]. According to a number of screenings, various classes of PCs are synthesized in rhododendron leaves, from phenolic acids to lignans and proanthocyanidins [34,35,36]. Phenylpropanoids are mainly represented by protocatechic, chlorogenic, and gallic acids, while caffeic and ellagic acids are species-specific [32,37]. Most representatives of the genus *Rhododendron* L. contain flavonols such as quercetin and its glycosides, in particular hyperoside and avicularin (3-O-galactoside and 3-O-arabinofuranoside of quercetin, respectively), whereas rutin (3-O-glucopyranoside of quercetin) was much less common [37,38]. The presence of 3-O-galactosides, 3-O-rhamnosides, and 3-O-arabinosides of kaempferol and myricetin, as well as 3-O-arabinoside of dihydroquercetin, 3-O-galactoside of gossypetin, 3-O-xyloside and 3-O-glucoside of quercetin, luteolin, and isorhamnetin, has been reported [39,40,41].

The application of high-performance liquid chromatography and mass spectrometry methods made it possible to identify some rare and species-specific metabolites of a phenolic nature in rhododendrons. For example, the flavanone matteucinol was found in the leaves of *R. simsii* and *R. dauricum*, which is extremely rare in higher plants [42,43], and two species-specific dihydroflavonols, trans– and cis-taxifolins, were found in *R. ferrugineum* [44]. Species-specific flavanones, decoroside A and B, have been identified in the leaves of *R. decorum* [45], and eight new lignans (rhomicranosides) were revealed in *R. micranthum* [34]. All this indicates a significant diversity of the rhododendrons’ phenolic complex.

Extracts obtained from plants of the genus *Rhododendron* L., as well as individual PCs, are successfully used for the prevention and treatment of various etiologies diseases. The antibacterial activity of ethanol and aqueous extracts of *R. ambiguum*, *R. tomentosum*, and *R. arboreum* containing naringenin, taxifolin, myricetin, and glycoside derivatives of p-coumaric and vanillic acids has been reported [22,46]. The high anti-inflammatory activity of rutin and taxifolin isolated from *R. luteum* and *R. arboretum* [22,41], cardioprotective activity of flavones of *R. simsii* [47], and the significant cytotoxic activity of cinnamantannin D1 (proanthocyanidin A-type trimer) from *R. formosanum* were noted [48].

The decrease in plant biodiversity, their habitat limitations and inaccessibility, as well as the negative effects of various anthropogenic factors, make it necessary to search for alternative ways to obtain medicinal plant biomass. These include biotechnological methods, such as in vitro culture initiation [49]. This also applies to microshoot cultures obtained through direct morphogenesis from plant explants under sterile conditions, which have genetic stability, a high level of cellular differentiation, and the ability to synthesize secondary metabolites, including PCs, characteristic of intact plants [49,50].

Currently, protocols have been developed for the in vitro cultivation of microshoots of some rhododendron species and their subsequent ex vivo adaptation [51,52,53,54,55]. There is little data on the accumulation of terpenoids [52] and PCs in them [56]. At the same time, very little is known about the content and composition of these secondary metabolites in the extracts of rhododendron microshoots, as well as their antioxidant activity [54,56,57].

Liposomal models are one of the modern approaches for assessing the effects of natural antioxidants, as well as studying biochemical processes in the cell membranes of living organisms [58,59]. They are also used as nanocontainers for the delivery of medicines, unsaturated omega-3 and omega-6 fatty acids, vitamins, etc. [60]. By initiating free radical chain reactions of oxidation of lipid components in the bilayer of cell membranes, it is possible to create a physiological model for assessing the antioxidant activity (AOA) of biologically active substances (BAS). In this regard, the search for BAS producers with high antioxidant potential is an urgent task in biology.

The aim of the study was to obtain in vitro microshoots of evergreen, semi-deciduous, and deciduous rhododendrons *(R. smirnowii*, *R. PGM Elite*, and *R. japonicum*, respectively). These genotypes are characterized by high adaptive potential, winter hardiness, and have been successfully introduced in most regions of Russia. The main objectives were to study their phenolic complex and antioxidant activity in ethanol extracts obtained from them. Such an approach will make it possible to evaluate the prospects of using rhododendron microshoots as the potential producers of plant bioantioxidants of a phenolic nature, which is a topical task of modern biotechnology and pharmacognosy.

## 2. Results

### 2.1. In Vitro Shoot Initiation, Proliferation, and Elongation of Rhododendron Microshoots

**Figure 1:**
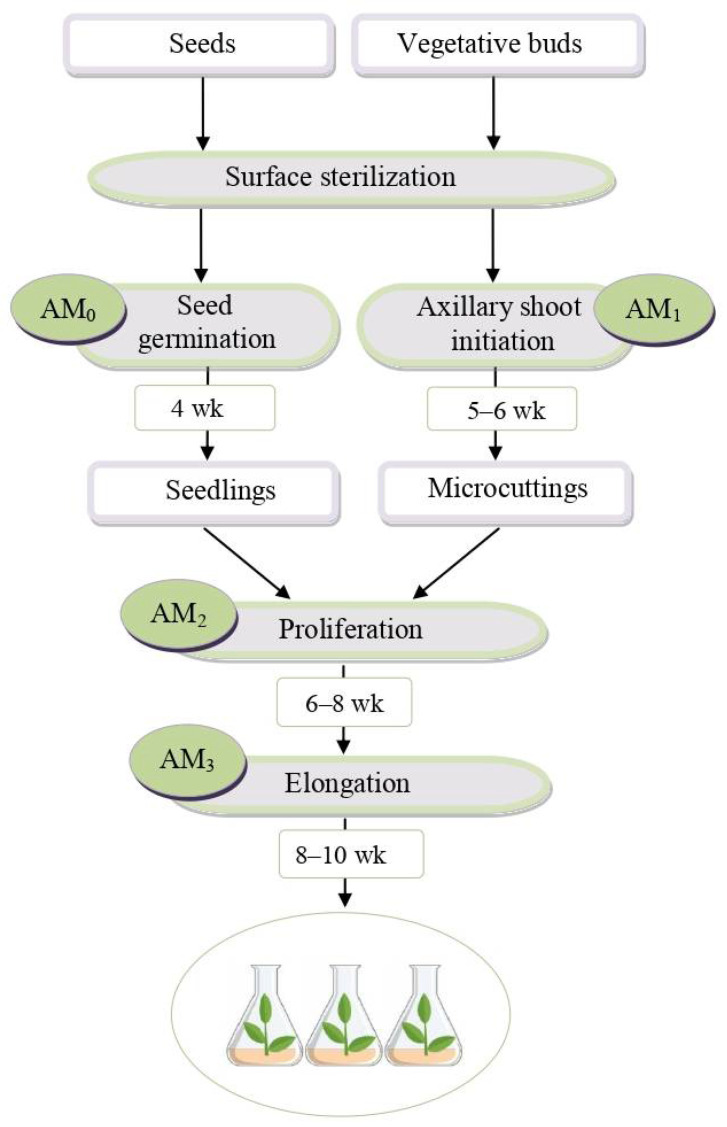
Flowchart of rhododendron micropropagation stages, excluding rooting and acclimatization. AM—basal Anderson’s medium: AM_0_—1/2 mineral nutrients, hormone-free; AM_1_—AM + 30–50 mg/L ascorbic acid + 0.5 mg/L IAA* + 2 mg/L 2iP**; AM_2_—AM + 4 mg/L IAA + 15 mg/L 2iP; AM_3_—AM + 1 mg/L IAA + 5 mg/L 2iP. *IAA—β-indoleacetic acid, **2iP—N^6^-2-isopentenyladenine.

**Figure 2:**
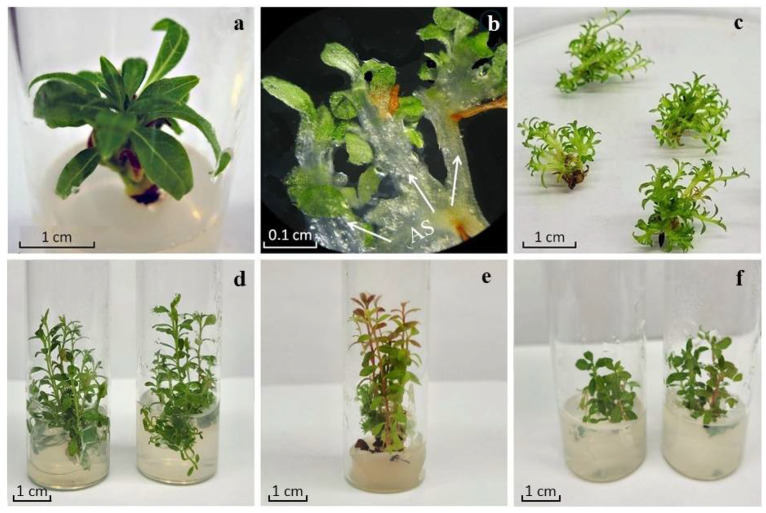
Micropropagation of rhododendrons: *R. PJM Elite* axillary shoots after 4 weeks of cultivation on AM_1_ initiation medium (**a**); initiation of adventitious shoots (AS) on *R. japonicum* explants (**b**); spherical conglomerates of *R. japonicum* microshoots after 10 weeks of cultivation on AM_2_ proliferation medium (**c**); *R. japonicum* microshoots (**d**), *R. PJM Elite* (**e**), and *R. smirnowii* (**f**) after 10 weeks of cultivation on AM_3_ elongation medium.

The introduction of rhododendrons into culture in vitro was carried out according to the protocol shown in Figure 1. For *R. japonicum* and *R. smirnowii*, 25-day-old seedlings obtained from seeds on a hormone-free nutrient medium AM_0_ were used as primary explants, whereas for *R. PJM Elite*, microcuttings of axillary shoots were obtained as a result of the precultivation of lateral and terminal buds on medium AM_1_ (Figure 2a).

To initiate proliferation, explants were inoculated onto the AM_2_ nutrient medium containing IAA and 2iP (ratio 1:4). After 4–5 weeks of cultivation, the formation of multiple adventitious buds was noted in the basal areas of the explants. Of these, by the sixth–seventh week of cultivation, all explants had the formation of strongly shortened de novo microshoots (Figure 2b). By the ninth week, the cultures were spherical conglomerates of 25–40 microshoots (Figure 2c). For elongation, microshoot conglomerates were divided into several parts, placed on an AM_3_ nutrient medium with a reduced hormone content, and grown for 8–10 weeks (Figure 2d–f). The obtained microshoots showed no signs of vitrification, which is an important indicator of the preservation of species and varietal characteristics in regenerating plants.

An assessment of the morphogenic potential of explants showed that in *R. japonicum* and *R. PJM Elite*, the regeneration frequency was almost equal and amounted to about 85–88% with the initial insertion of 6–9 buds per explant, whereas in *R. smirnowii*, it was 65% with 5–6 buds per explant. This indicates a low morphogenic potential of the explants of the evergreen *R. smirnowii* in vitro compared with *R. japonicum* and *R. PJM Elite*.

### 2.2. Morphophysiological Characteristics of Rhododendron Microshoots

Microshoots of three *Rhododendron* species at the elongation stage differed in morphometric parameters (Table 1).

In *R. smirnowii*, the length of shoots, their raw mass, and the number of internodes had the lowest values. In *R. japonicum* and *R. PJM Elite*, they were equal to each other. Significant differences have been identified in the branching patterns of the microshoots of all species. Thus, in *R. japonicum* it was observed in 25% of microshoots, in *R. PJM Elite*—in individual cases, in *R. smirnowii*—it was completely absent. Consequently, *R. japonicum* was characterized by active branching of microshoots in comparison with other studied species.

The water content in the microshoot tissues of all variants had similar values. At the same time, it was equal in *R. japonicum* and *R. PJM Elite* and slightly, but statistically significantly lower in *R. smirnowii*.

### 2.3. The Photosynthetic Pigment Content in Rhododendron Microshoots

The determination of chlorophyll *a* and *b* content (Chl *a*, Chl *b*) in the microshoots of three *Rhododendron* species demonstrated significant differences between them (Table 2). *R. PJM Elite* is characterized by the maximum accumulation of photosynthetic pigments. In *R. smirnowii* and *R. japonicum*, it was lower by 33% and 42%, respectively. This concerned both the content of Chl *a* and Ch *b* and their total content (*a* + *b*). At the same time, the Chl *a* content in all variants was almost 2 times higher than that of Chl *b*.

The ratio of chlorophyll *a/b* was equal in the microshoots of *R. PJM Elite* and *R. japonicum*, whereas in *R. smirnowii* it was 20% higher.

### 2.4. The Phenolic Compound Content in Rhododendron Microshoots

**Figure 3:**
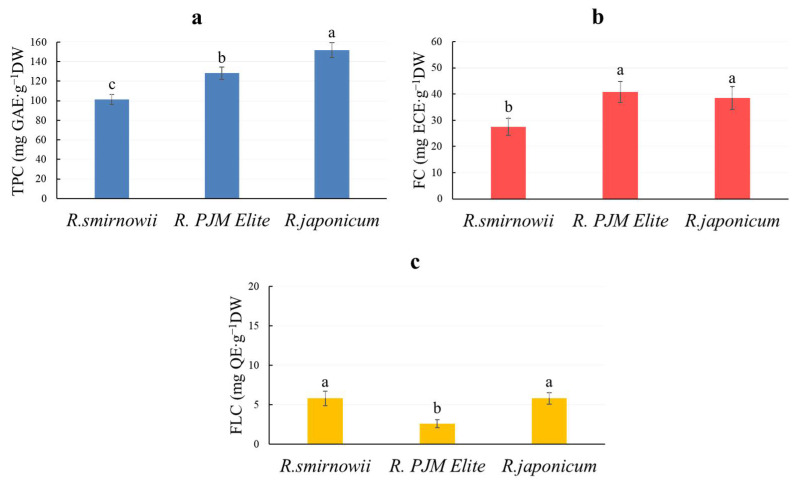
The total phenolic content—TPC (**a**), flavanol content—FC (**b**), and flavonol content—FLC (**c**) in microshoots of various *Rhododendron* species. Results are expressed as means ± SD, *n* = 3. The significant differences at *p* < 0.05 are indicated by different Latin letters.

PCs are one of the most common secondary metabolites in plant tissues [1]. Total phenolic content (TPC) was highest in the microshoots of *R. japonicum*, exceeding that of *R. smirnowii* and *R. PJM Elite* by 40% and 20%, respectively (Figure 3a).

PCs characteristic of plants of the genus *Rhododendron* L. include flavonols and flavanols [37,38]. The content of flavonols in microshoots of *R. smirnowii* and *R. japonicum* was equal and almost twice that of *R. PJM Elite* (Figure 3c). A different trend is typical for flavanols (Figure 3b). Their accumulation was almost the same in microshoots of *R. japonicum* and *R. PJM Elite*, exceeding that of *R. smirnowii* by 25%.

### 2.5. Phenolic Profile of Rhododendron Microshoots

The study of the phenolic complex of microshoot ethanol extracts of different *Rhododendron* species using high-performance liquid chromatography (HPLC) revealed significant differences between them (Table S1 and Figure S1). In the extracts of *R. smirnowii* and *R. japonicum*, 20 phenolic compounds were found, the total content of which was 9.14 and 12.75 mg/gDW, respectively. In *R. PJM Elite*, the composition was less diverse and was represented by 12 compounds, the content of which was 7.75 mg/gDW. In all variants, the dominant components among the identified PCs were flavonoids, mainly flavonols, present both as aglycones and glycosides.

Since the identification of flavonol, namely flavonol glycosides, in native extracts is difficult, their content was determined by aglycones (quercetin, kaempferol, and myricetin), formed after the acid hydrolysis of the corresponding glycosides [61,62].

According to the obtained data, all rhododendron microshoots were characterized by a high content of quercetin glycosides, which included monosaccharides such as D-glucose, D-galactose, L-arabinose, and L-rhamnose. In *R. japonicum,* it was maximum and twice as high as in *R. PJM Elite* and *R. smirnowii*. Kaempferol glycosides (mainly gluco- and rutinosides) were found in the extracts of *R. japonicum* and *R. smirnowii*, and myricetin glycosides were found in the extracts of *R. japonicum* and *R. PJM Elite*, but in very low quantities.

**Figure 4:**
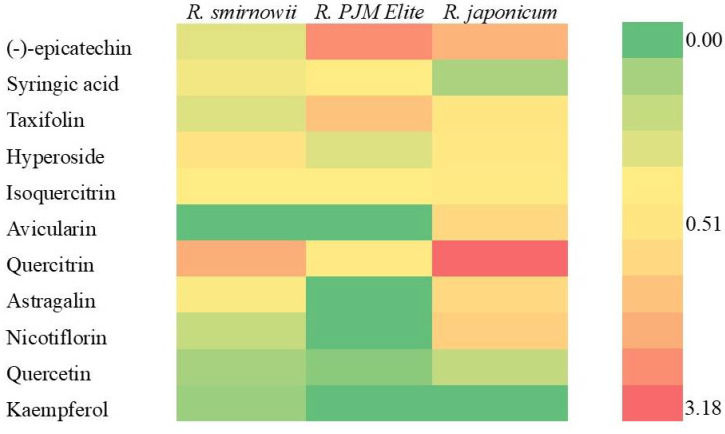
The identified phenolic compound content (mg/gDW) in microshoots of various *Rhododendron* species.

At the same time, there were interspecific differences in the composition of individual components of the flavonol complex (Figure 4). Thus, in *R. japonicum*, quercitrin (quercetin-3-O-α-L-rhamnoside), nicotiflorin (kaempferol-3-O-rutinoside), and avicularin (quercetin-3-O-α-L-arabinoside) predominated; in *R. smirnowii*, quercitrin and hyperoside (quercetin-3-β-D-galactoside); in *R. PJM Elite*, quercitrin and isoquercitrin (quercetin-3-O-glucopyranoside).

Along with flavonols, other compounds of a phenolic nature were identified in extracts of rhododendron microshoots (Table S1, Figure 4). Flavanol-(−)-epicatechin and flavanonol—taxifolin (2,3-dihydroquercetin) were present in all variants. Their greatest accumulation is typical for the microshoots of *R. PJM Elite* and *R. japonicum*. In addition, syringic acid, belonging to the class of phenylpropanoids, has been identified. Its highest content was noted in *R. PJM Elite* and *R. smirnowii*, whereas it was significantly lower in *R. japonicum*.

A number of PCs have not been identified; however, based on their chromatographic characteristics, it can be assumed that compounds 3, 5, and 6 belong to oxybenzoic acids, while compounds 21, 22, and 24–27 belong to flavonols (Table S1, Figure S1).

**Figure 5:**
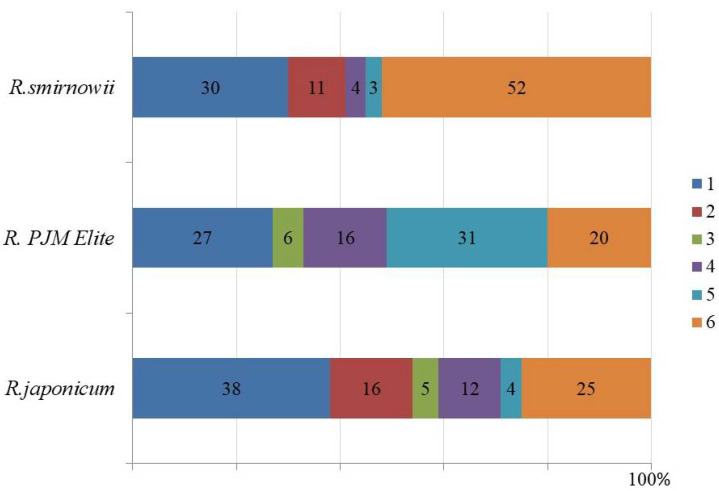
Balance of the main phenolic compounds in microshoots of various *Rhododendron* species (%): quercetin glycosides (1); kaempferol glycosides (2); myricetin glycosides (3); (−)-epicatechin (4); taxifolin (5); others (6).

According to the obtained data, the widest spectrum and the highest content of PCs were characteristic of microshoots of *R. japonicum*, where the dominant components were flavonols, represented by glycosides of quercetin, kaempferol, and myricetin. Their ratio in the total content of PCs in *R. japonicum* was about 60%, while in *R. smirnowii* and *R. PJM Elite*, it was 40 and 33%, respectively (Figure 5).

In the microshoots of *R. PJM Elite*, the balance of individual compounds differed significantly from that of other species, which may be due to the high proportion of taxifolin (31%) and epicatechin (16%), as well as the low proportion of flavonols, including the absence of kaempferol glycoside.

### 2.6. Antiradical Activity of Rhododendron Microshoot Extracts in the DPPH-Radical System

The method we used to determine the antiradical activity (ARA) of plant extracts is based on the process of reducing the free stable chromogenic radical 2,2-diphenyl-1-picrylhydrazyl (DPPH) with an antioxidant (AO), which depends on the pH of the reaction medium and the qualitative composition of the extract [63,64].

In this work, 80% ethanol was used as an extractant of phenolic antioxidants and a solvent for DPPH, since the use of other extractants (water, acetone) in some cases led to a significant decrease in ARA [64,65]. As a result of determining the dependence of ARA on the concentration of ethanol extracts of rhododendron microshoots, their optimal values were found to be 30–33.3 mg_dw_/mL (Figure S2). For concentrations above 40–50 μg_dw_/mL, after 30 min of exposure, the curve of the dependence of DPPH-radical inhibition became nonlinear and reached saturation.

**Figure 6:**
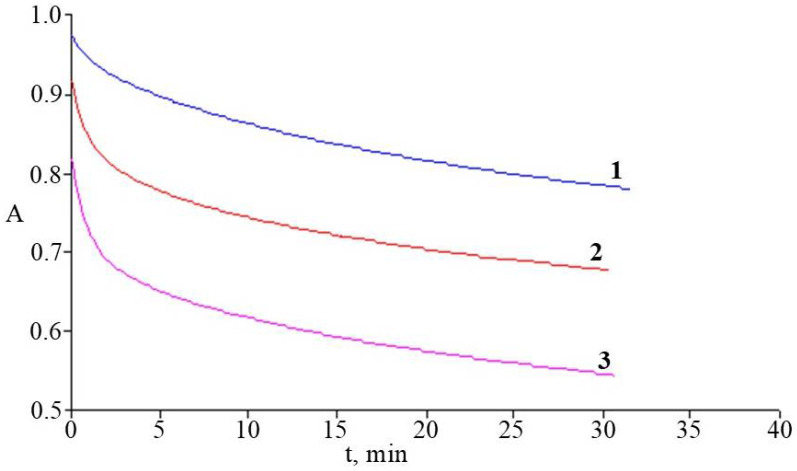
Kinetics of DPPH radicals’ recovery by antioxidant components of microshoots’ extracts: *R. smirnowii* (**1**), *R. PJM Elite* (**2**), *R. japonicum* (**3**). A—optical density of DPPH solution at λ = 520 nm. Concentration of the 80% ethanol extracts is 33.3 μg_dw_/mL.

The study of the change pattern in the kinetic curves of the DPPH radical’s recovery by the antioxidant components of rhododendron microshoots’ ethanol extracts showed that their introduction into the reaction mixture was accompanied by a dramatic decrease in the optical density of the sample (Figure 6). This trend was most characteristic of the *R. japonicum* extract, which is probably due to the highest content of phenolic AO in it, with a predominance of compounds with a high rate constant of interaction with the radical.

The main kinetic parameters of the ARA assessment of microshoots’ ethanol extracts of three *Rhododendron* species are shown in Table 3.

According to the data obtained, the ARA of extracts increased in the range of *R. smirnowii* < *R. PJM Elite* < *R. japonicum*. At the same time, the extracts of *R. PJM Elite* maintained a high ARA despite the lowest level of PCs accumulation compared to other species.

### 2.7. Antioxidant Activity of Rhododendrons’ Extracts in the System of Initiated Liposome Oxidation

The system of 2,2′-azobis-(amidinopropane)-dihydrochloride (AAPH)-initiated oxidation of phosphatidylcholine liposomes (PCh-liposomes) and its inhibition by AO of plant extracts is complex, but it is the most realistic physiological model for studying the mechanisms of oxidative damage to the cell membranes of living organisms [58,66,67].

In our study, the main kinetic parameter for evaluating plant extracts APA was the induction period τ, reflecting the time from the beginning to the point of oxidation inhibition (τ_0_ was 5 ± 0.5 min). In addition, to determine the concentration dependences of the inhibitory effect of extracts, the parameter C was used, reflecting the specific content of the microshoots’ dry mass in a certain aliquot of extract (m_dw_, mg) introduced into the liposome suspension to the mass of PCh (m_PCh_, mg):

C = m_dw_/m__PC__h__

In the range of specific C content from 0.002 to 0.012 mg/mg, linear dependences τ(C) were obtained, the coefficient (slope) of which expressed the AOA of the studied extracts (Figure S3). The results of measuring the dependence of the liposome oxidation inhibition time on C and the values of their antioxidant activity, expressed in the trolox equivalent (AOA), are shown in Table 4.

**Figure 7:**
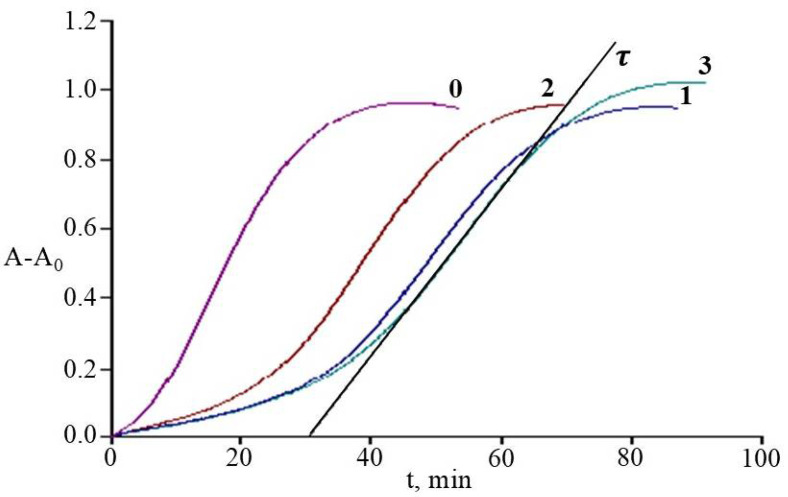
Kinetic curves of increase in optical density (A-A_0_) at λ = 234 nm (formation of DC) in the process of initiated oxidation of liposomes without introducing extracts (**0**); with added extracts *R. smirnowii* (**1**), *R. PJM Elite* (**2**), *R. japonicum* (**3**). APPH concentration = 0.33 µM; C = 0.06 μg_dw_/mg_PCh_; T = 37 °C; A_0_ is the optical density of DC at t = 0; τ-induction period.

Figure 7 shows the comparative kinetic curves of the formation of diene conjugates (DC) during liposome oxidation without and with the addition of rhododendron microshoots’ extracts to the suspension at the same value of their specific content (C).

All the studied extracts showed their effectiveness in inhibiting liposome oxidation processes, varying in the range *R. PJM Elite* < *R. smirnowii* < *R. japonicum*. Extracts of *R. japonicum* and *R. smirnowii* showed the greatest AOA, characterized by a high total content of phenolics and flavonols with significant differences in the composition of their phenolic complex.

## 3. Discussion

### 3.1. In Vitro Shoot Initiation, Proliferation, and Elongation of Rhododendron Microshoots

In vitro cultivation is widely used to preserve and propagate plants with a limited range of natural distribution and high pharmacological potential [49]. This approach reduces the pressure on wild plant populations and ensures the year-round availability of plant biomass. The optimization of cultivation conditions, cell selection, the application of elicitors and other exogenous factors, including hormonal ones, can create a good prospect for the scalable and controlled production of pharmacologically valuable plant metabolites that are essential for public health. However, it is necessary to take into account a number of potential problems, namely, the economically justified choice of the object, the cost of its cultivation and production, and the bioavailability of BAS. Currently, certain successes have been achieved in the clonal micropropagation of medicinal plants such as *Hypericum perforatum* L., *Rhodiola rosea* L., *Schisandra rubriflora*, and many others [68,69,70,71], while the production and cultivation of rhododendron microshoots as potential producers of pharmacologically valuable bioflavonoids has been studied very little [52,54,56,57].

The clonal micropropagation of *R. smirnowii*, *R. japonicum*, and *R. PJM Elite* was performed according to the protocol shown in Figure 1 using 2iP, a synthetic cytokinin group hormone, as an inducer of morphogenesis [53]. It makes it possible to obtain regenerating plants that develop through direct organogenesis, bypassing the stage of callus formation, which ensures the preservation of the genetic stability of the propagated forms [72]. A number of studies have noted the promise of using 2iP in the micropropagation of the plants of the Ericaceae DC in comparison with thidiazuron, which often causes vitrification and other anomalies in the morphology of microshoots [73,74].

### 3.2. Morphophysiological Characteristics of Rhododendrons Microshoots

The study of in vitro cultures morphophysiological characteristics is an important indicator of their physiological status and differentiation level.

According to our data, the formation of adventitious microshoots occurred mainly in the basal part of rhododendron explants cultivated on a proliferation medium (Figure 2). Their subsequent growth on the elongation medium was accompanied by the formation of elongated microshoots of normal morphological structure (Figure 2d–f). The length, mass, and branching pattern differed in the studied variants (Table 1). At the same time, the species-specific features inherent in intact rhododendron plants were preserved in vitro. So, microshoots of *R. smirnowii* were characterized by the lowest growth rate and complete absence of branching compared to other species (Figure 2f). To a certain extent, this is consistent with the ontogenetic characteristics of intact plants. It is known that in slow-growing evergreen rhododendrons, which include *R. smirnowii*, branching usually occurs only for 5–7 years of vegetation, whereas in deciduous species (in our case, *R. japonicum*), it occurs for 2–3 years [28,29]. This once again confirms the important role of the genotype in the clonal micropropagation of plants in vitro [70,75].

### 3.3. The Photosynthetic Pigment Content in Rhododendron Microshoots

The growth, development, and productivity of plants are determined by the efficiency of the photosynthesis process, the indicators of the functional activity of which are the content of pigments such as Chl *a* and Chl *b*, as well as their ratio [76]. According to our data, their accumulation in the microshoots of the three *Rhododendron* species was different (Table 2). The maximum accumulation is typical for *R. PJM Elite*, the minimum for *R. japonicum*. At the same time, the differences between them did not exceed 40%. Since the level of insolation during the cultivation of rhododendron microshoots under in vitro conditions was the same, these differences are probably due to their morphophysiological characteristics. Therefore, for *R. PJM Elite*, characterized by rapid shoot growth and a large number of internodes and foliage (Table 1), chlorophyll accumulation was higher compared to other species (Table 2).

An important indicator in assessing the photosynthetic ability of plant tissues is the Chl *a/b* ratio [76,77]. Its greatest value was found in microshoots of the evergreen *R. smirnowii* relative to two other species, in which it was equal and 22% lower. This may be a consequence of the ecophysiology of evergreen, shade-tolerant plants preserved in vitro, as well as the activity of the photosystem II light-harvesting complex [30,78].

All these data once again confirm the dependence of the content and ratio of chlorophylls, which play a key role in the functioning of photosynthesis, and also of the plants’ species, their growing conditions, and features of ontogenesis [77,79].

### 3.4. The Phenolic Compounds Content in Rhododendron Microshoots

As mentioned above, in vitro cultures are promising sources of biologically active substances, including PCs, successfully used in medicine and pharmacology. They retain and sometimes surpass intact plants in the ability to accumulate these metabolites [69,80,81]. In particular, a high content of PCs was noted in in vitro cultures with a high level of differentiation—hairyroots and microshoots [80]. However, data on the accumulation of these secondary compounds in rhododendron microshoots are scarce and contradictory [52,54,56].

The determination of the total phenolic content is considered one of the important indicators for assessing the potential ability of plants in relation to the biosynthesis of these secondary metabolites [82,83]. In our case, the greatest ability to accumulate them is noted in the microshoots of *R. japonicum*. It was lower for *R. PJM Elite* and *R. smirnowii* by 15 and 20%, respectively (Figure 3a). It should be noted that the PCs content in the rhododendron microshoots was two times lower compared to intact plants [35,36,37], which is often observed by other researchers for various in vitro cultures. At the same time, in most cases, the preservation of species-specific features of phenolic metabolism is noted.

It is known that rhododendrons are characterized by the formation of PCs such as flavonols and flavanols [38]. At the same time, flavonol biosynthesis is carried out at earlier stages of phenolic metabolism relative to flavanols [3]. According to our data, the microshoots of the three *Rhododendron* species differed in the content of these PCs (Figure 3b,c). At the same time, in *R. PJM Elite,* the amount of flavonols was significantly lower compared to other species, while the amount of flavanols was higher.

Consequently, the revealed features of the main PCs classes’ balance in microshoots of the semi-deciduous hybrid form *R. PJM Elite* reveal differences in the direction of their biosynthesis compared with deciduous *R. japonicum* and especially with evergreen *R. smirnowii*. All this indicates the preservation of in vitro metabolic features of intact plants, which has been repeatedly noted by various authors [11]. An important role of chloroplasts as one of the sites of PCs biosynthesis in plant cells cannot be excluded [84,85]. As we noted above, the maximum content of Chl *a* and Chl *b* was noted in the microshoots of *R. PJM Elite* relative to other species (Table 2). This once again confirms the relationship between the photosynthetic activity of plant tissues and the accumulation of PCs in them.

### 3.5. Phenolic Profile of Rhododendron Microshoots

The determination of the various PCs classes’ content in plant material involves assessing only their total level, without clarifying the contribution of individual representatives. In this regard, the next stage of the work was to study the metabolic profile of these substances using the HPLC method. According to the data obtained, the microshoots of rhododendrons at the final stages of the elongation stage differed in the content of PCs in the series *R. PJM Elite* < *R. smirnowii* < *R. japonicum* in the range from 7.75 to 12.75 mg/g of dry weight. This indicates a higher ability of the microshoots of deciduous *R. japonicum* to accumulate these secondary metabolites compared with other species (Table S1), which is consistent with the data on the total content of PCs obtained by the spectrophotometric method.

The study of phenolic metabolites in microshoots of various *Rhododendron* species revealed both similarities and differences in their composition and quantity (Table S1, Figure 4). All cultures are characterized by the formation of syringic acid, which belongs to oxybenzoic acids, the most biogenically early compounds of phenolic metabolism [1,3]. At the same time, chlorogenic and neochlorogenic acids, characteristic of the aboveground organs of rhododendrons grown in vivo, were not detected [36,37].

The main components of the phenolic complex of rhododendron microshoots were flavonoids, the most numerous class of PCs in higher plants [14,15]. Their spectrum is extremely diverse, and they are represented not only by aglycones but also by glycosides (mainly flavonol glycosides). (−)-Epicatechin, taxifolin, hyperoside, isoquercitrin, quercitrin, and quercetin were found in all variants (Table S1). There were also a number of significant species-specific differences in the phenolic profile in microshoots of various *Rhododendron* species. Therefore, kaempferol and its glycosides were not detected in *R. PJM Elite*, and the level of taxifolin and (−)-epicatechin was two to three times higher than that of other species. Avicularin was found only in extracts of *R. japonicum*, characterized by a high content of quercetin and its derivatives. It should also be noted that rutin, the accumulation of which is typical for intact plants, was not detected in microshoots of all studied *Rhododendron* species [37,38]. This may be due to the functional role of rutin, which is actively synthesized at the stage of rooting and acclimatization of microshoots ex vivo [56].

All this indicates differences in the composition of the phenolic profile main components of the various *Rhododendron* species microshoots. In *R. smirnowii* and *R. japonicum*, flavonols were the dominant PCs, whereas in *R. PJM Elite,* along with them, the proportion of flavanols was also high (Figure 5). Consequently, in the semi-deciduous *R. PJM Elite*, PCs biosynthesis is directed towards the formation of (−)–epicatechin and taxifolin, pharmacologically valuable plant metabolites, which are of interest for further research.

### 3.6. Antiradical Activity of Rhododendron Microshoot Extracts in the DPPH-Radical System

It is known that PCs, mainly of a flavonoid nature, belong to the low-molecular-weight component of the antioxidant protection system of plant tissues. This is due to their redox potential, which makes it possible to act both as hydrogen donors and as singlet oxygen scavengers, as well as chelators of transition metal ions [14,15]. AOA and ARA of the PCs of different classes have been reported in various model systems, as well as an analysis of the dependence of their structure/activity [9,86,87]. At the same time, flavonoid glycosides were characterized by lower ARA values relative to aglycones [9].

To analyze the ARA of rhododendron extracts, a model of DPPH radical reduction was used [10]. It should be noted that this reaction in polar ionizing solvents (ethanol, methanol) can proceed by two parallel mechanisms: Electron Transfer–Proton Transfer (ET–PT) and Sequential Proton Loss Electron Transfer (SPLET), with a pronounced dominance of the latter [63,64].

According to the data obtained, the ARA of microshoots’ ethanol extracts was the highest in *R. japonicum*, whereas in *R. PJM Elite* and *R. smirnowii*, it was lower by 20% and 30%, respectively (Figure 6, Table 3). These differences may be due to the composition of phenolic AO, as well as the rate constants of their interaction with the radical.

For all the samples studied, a high correlation (R^2^ = 0.997) was observed between the ARA of the extracts and flavonoid content in them. Thus, extracts of *R. japonicum* and *R. PJM Elite* showed the greatest ARA, containing a high proportion of taxifolin, as well as quercetin and myricetin derivatives. At the same time, the absence of a highly effective AO kaempferol (both aglycone and its glycosides) in the phenolic profile of *R. PJM Elite* extracts was not accompanied by a decrease in ARA (Figure 4). Thus, in the reduction reaction of the DPPH radical, which is mainly proceeded by the SPLET mechanism. The greatest effectiveness of the rhododendron microshoot extracts may be due to the action of quercetin and myricetin derivatives. This is due to the number and stereochemical position of hydroxyl groups in the B-ring of these PCs [87]. Taxifolin makes a much smaller contribution to the ARA of extracts, which is probably due to the absence of a C2-C3 double bond despite the presence of a catechol fragment [4,14].

### 3.7. Antioxidant Activity of Rhododendrons’ Extracts in the System of Initiated Liposome Oxidation

A model of initiated oxidation of PCh-liposomes was used to analyze the AOA of rhododendron microshoot extracts. Most often, in organic solvents (ethanol, methanol), phenolic AOs interact with the radical, mainly through the mechanism of Hydrogen Atom Transfer (HAT), or they affect the rate of lipid oxidation due to the formation of membrane-associated complexes and changes in membrane fluidity [58,66]. At the same time, the more hydrophobic phenolic AOs are implemented in the lipid core of the membrane, while the hydrophilic ones form hydrogen bonds with the polar heads of the phospholipid bilayer at the lipid–water interface. Both types of interactions can provide a certain level of protection against the damaging effects of reactive oxygen species and contribute to the stabilization and preservation of the biological membranes’ function [58,66,67].

According to our data, the AOA of rhododendron microshoot extracts in the system of initiated PCh-liposome oxidation correlated with the total content of PCs and flavonols in them (R^2^ = 0.952 and R^2^ = 0.958, respectively). The greatest AOA was shown by extracts of *R. japonicum*, which had a high content of quercetin and kaempferol derivatives. Similar kinetics of liposome oxidation inhibition were shown by *R. japonicum* and *R. smirnowii* extracts, despite the significant differences in their phenolic profile (Figure 7). This may be due to both the synergism and antagonism of PCs plant extracts, as well as their pro-oxidant effect [9,88,89].

## 4. Materials and Methods

### 4.1. Plant Material and Cultivation Conditions

The object of the study was the in vitro microshoots of the plants of the genus *Rhododendron* L: evergreen *R. smirnowii* Trautv, deciduous *R. japonicum* (A.Gray), and semi-deciduous *R. PJM Elite* (hybrid form of *R. carolinianum* × *R. dauricum* var. *sempervirens*).

To introduce *R. smirnowii* and *R. japonicum* into culture, seeds obtained from the seed bank of N.V. Tsitsin’s Botanical Garden RAS (Moscow, Russia) were used, and for *R. PJM Elite*, the vegetative terminal and lateral buds of the annual shoots of plants growing in open ground in the arboretum of N.V. Tsitsin’s Botanical Garden RAS (Moscow, Russia) were used.

All the cultures were maintained in the growth cabinet at 25 °C and 80% relative humidity (RH) under conditions of 16 h photoperiod with 50 µmol m^−2^ s^−1^ photosynthetic photon flux density (PPFD) provided by cool white fluorescent light (40 Wtubes, Philips, Eindhoven, The Netherlands).

### 4.2. In Vitro Culture Initiation

Seeds of *R. smirnowii* и *R. japonicum* were decontaminated with 80% (*v*/*v*) ethanol for 30 s followed by 1.0% (*v*/*v*) sodium hypochlorite (NaClO) (Produits Dentaires SA, Vevey, Switzerland) for 4 min. Vegetative terminal and lateral buds of *R. PJM Elite* treated with 7% Fundazol solution (Agro-Chemie, Budapest, Hungary) at an exposure of 20 min, 70% ethanol for 10 s, and 2% mercuric chloride (Merck, Darmstadt, Germany) for 8–10 min were applied sequentially. After sterilization, the seeds and buds were washed with sterile distilled water 7–8 times under aseptic conditions.

Sterilized rhododendron seeds were placed on a hormone-free nutrient medium Anderson [90] with half-reduced mineral composition, 3% (*w*/*v*) sucrose, and 0.8% (*w*/*v*) agar (medium AM_0_). The duration of germination was 4 weeks.

When using *R. PJM Elite* buds as initial explants, the bud scales were removed from them under aseptic conditions and placed on a modified Anderson nutrient medium containing 30–50 mg/L ascorbic acid, 0.5 mg/L β-indoleacetic acid (IAA; Sigma–Aldrich, Saint Louis, MO, USA), and 2mg/L N^6^-2-Isopentenyladenine (2iP; Sigma–Aldrich, USA) (medium AM_1_) [53]. The duration of the cultivation for the initiation of axillary shoots was 5–6 weeks.

### 4.3. In Vitro Shoot Proliferation

To initiate proliferation, fragments of 25-day-old in vitro seedlings of *R. smirnowii* and *R. japonicum* (without hypocotyl and cotyledon leaves), as well as microcuttings of axillary shoots of *R. PJM Elite*, were used by placing them on a modified Anderson nutrient medium with the addition of 4.0 mg/L IAA and 15.0 mg/L 2iP (medium AM_2_) [53]. The duration of cultivation was 6–8 weeks.

### 4.4. In Vitro Shoot Elongation

Spherical conglomerates of microshoots obtained at the proliferation stage were divided into several parts and transferred to a modified Anderson nutrient medium with a reduced hormone content (1.0 mg/L IAA and 5.0 mg/L 2iP) (AM_3_ medium) for elongation [53]. The duration of cultivation was 8–10 weeks.

### 4.5. Morphometric Analysis

The fresh weight of rhododendron microshoots, as well as their length, number of internodes, and branching frequency, were assessed. A representative sample consisted of 20 plants at the final stages of elongation (8–10 weeks).

### 4.6. Determination of Water Content

A sample of rhododendron microshoots (150 mg) was dried in a BD-115 thermostat (Binder, Tuttlingen, Germany) at +70 °C to constant weight. Water content was calculated using the standard method [91].

### 4.7. Determination of Chlorophyll a and b Content

To extract photosynthetic pigments, rhododendron microshoots were crushed and extracted with 96% ethanol [77,92]. The homogenate was centrifuged at 13,000× *g* (centrifuge Minispin, Gottingen, Germany) and the supernatant was used for the spectrophotometric determination of chlorophyll *a* and *b* content at 665 and 649 nm, respectively [93]. The pigment concentration was calculated based on specific absorption coefficients and expressed as mg/g of fresh weight [94].

### 4.8. Determination of Different Phenolic Compounds Classes Total Content

Phenolic compounds were extracted with 96% ethanol from freshly crushed plant material at 45 °C for 45 min. The homogenate was centrifuged (16,000 rpm, 15 min), and the supernatant was used for spectrophotometric studies. The content of total PCs was determined with Folin–Ciocalteu reagent (725 nm), according to the method described by us earlier [95]. The determination of the total flavonols was carried out with 5% aluminum chloride (415 nm) [96]. To determine flavanols, 1% vanillin reagent (500 nm) was used [97].

The total phenolic content was expressed in mg of gallic acid equivalents per g of dry weight (mg GAE∙g^−1^ DW), the content of flavonols in mg of quercetin equivalents per g of dry weight (mg QE∙g^−1^ DW), the content of flavanols in mg of epicatechin equivalents per g of dry weight (mg ECE∙g^−1^ DW).

### 4.9. Analysis of Individual Phenolic Compounds Using High Performance Liquid Chromatography (HPLC)

Microshoots of different rhododendrons cultivated in the elongation stage for 9–10 weeks were used for analysis. To prepare the extracts, the plant material was lyophilized for 48 h at a pressure of 3–15 Pa (Liovak GT2, Leybold-Heraus, Hanau, Germany). After that, the dry material was shredded into 2–3 mm pieces and blended. Representative samples were extracted with 70% ethanol in a water bath. The supernatant was separated by filtration. The resulting eluate was passed through a Diapak C16 concentrating cartridge (BioChemMack, Moscow, Russia), washed with 70% ethanol, and then passed through a membrane filter with a pore diameter of 0.45 μm [98].

The analysis of phenolic compounds was performed by means of an Agilent 1200 HPLC system equipped with a diode array detector and a ChemStation system for the recording and processing of chromatographic data (Agilent Technology, Santa Clara, CA, USA). The chromatographic separation was performed on a Zorbax SB-C18 column (5 μm, 4.6 × 150 mm) at 25 °C. Methanol content of the mobile phase in an aqueous solution of phosphoric acid (0.1%) varied from 31 to 33%, from 46 to 56%, and from 56 to 100% during 4 min (system 1). The eluent flow rate was 1 mL/min. Detection wavelengths were 254, 270, 290, 340, 360, and 370 nm, and classes of phenolics were identified by their spectral characteristics [98,99]. For the identification of the phenolics in the plant extracts, we used standard samples of cinnamic and caffeic acids (Serva, Heidelberg, Germany), taxifolin (Austrowaren, Wien, Austria), chlorogenic and p-coumaric acids, quercetin, kaempferol (Sigma–Aldrich, Steinheim, Germany), isoquercitrin, rutin, avicularin, astragalin, and hyperoside (FlukaChemie AG, Buchs, Switzerland) in a concentration of 10 µg/mL. Quantification of individual components in the plant samples was performed with an external standard method [61]. Concentrations of unidentified flavonoids were calculated by means of hyperoside, and those of unidentified acids were computed by means of chlorogenic acid.

Determination of the flavonol glycosides’ content was carried out by analysis of free aglycones formed after acid hydrolysis of the corresponding glycosides, followed by conversion to flavonol glycosides [61,62,98]. Methanol content of the mobile phase in an aqueous solution of phosphoric acid (0.1%) varied from 45 to 48% during 18 min [61], and the eluent flow rate was 1 mL/min (system 2). Detection wavelengths were 255, 270, 290, 340, 360, and 370 nm. In the hydrolysates of extracts, the concentration of all substances is calculated using quercetin. To recalculate the concentration of aglycone to the corresponding glycoside, the coefficients were used—2.504 for quercetin and myricetin, and 2.588 for kaempferol [61].

The relative standard deviation of repeatability in the determination of phenolics was σr, rel = 0.011, and the relative standard deviation of retention time in HPLC analysis was 0.0018.

### 4.10. Determination of the Antiradical Activity of In Vitro Rhododendron Microshoots’ Extracts in the 1,1-Diphenyl-2-picrylhydrazyl Radical System

The antiradical activity (ARA) of in vitro extracts of rhododendron microshoots was determined by reaction with the stable chromogen-radical—2,2-diphenyl-1-picrylhydrazyl (DPPH) [100]. To do this, different volumes of extracts obtained from microshoots of different types of rhododendrons were added to 1 mL of 80% ethanol solution of DPPH at a concentration of 0.2 mM in the working cuvette. The total volume was adjusted with 80% ethanol to 2 mL and mixed. The kinetics of decreasing the optical density of the DPPH (A_0_) solution was recorded for 30 min on a PerkinElmer spectrophotometer (Bruker, Germany) at 520 nm. The comparison cell contained 80% ethanol. The optical density of the control solution of DPPH (A_0_), which did not contain the extract, was approximately 1.2 and varied within 0.5% for 30 min.

The efficiency of DPPH-radical recovery (inhibitory activity, I) by components of rhododendron extracts was calculated using the formula:

I, % = [(A_0_ − A_30_)/(A_0_)] 100

The ARA value was expressed in trolox equivalents (Tr_eq_., µmolTr/mg) using the regression equation of the dependence of I on the concentration of Tr:

y = 2.2623x + 2.3226 (R^2^ = 0.978)

Calibration curves for 80% ethanol solutions of trolox were constructed in the concentration range from 5 to 40 mM. For each concentration, measurements were carried out at least 3 times, and their error did not exceed ±6%.

### 4.11. Determination of the Antioxidant Activity of In Vitro Rhododendron Microshoots’ Extracts in the System of Initiated Liposome Oxidation

The antioxidant activity (AOA) of extracts obtained from in vitro rhododendron microshoots was determined using a model system of initiated liposome oxidation from soy phosphatidylcholine (PCh) [58,88,101]. To prepare liposomes, a suspension of soy PCh (L-α-phosphatidylcholine P3644, Sigma–Aldrich, USA) was used in a 1 mM phosphate buffer with a pH of 7.4. For this purpose, it was mixed in a shaker (20 min), adding different volumes (from 3 to 30 µL) of ethanol extracts of rhododendron microshoots. Liposomes were formed using a VCX-130 ultrasonic homogenizer (Sonics&Materials, Newtown, CT, USA) with constant cooling of the suspension vessel to 0 °C, which prevented PCh oxidation. The resulting liposome suspension was centrifuged (12,000× *g*, 20 min) at +4 °C, followed by the selection of a supernatant.

To initiate liposome oxidation, a water-soluble azo-initiator, 2,2′-azobis-(amidinopropane)-dihydrochloride (AAPH) (Fluka, Steinheim, Germany) with a final concentration of 0.33 mM in liposome dispersion, was used. Liposome oxidation with a PH concentration of 0.1 mg/mL was performed in quartz cuvettes thermostated at a physiological temperature of +37 °C on a Lambda-25 spectrophotometer (PerkinElmer, Germany). The kinetics of formation of lipid peroxidation (LPO) products—diene conjugates (DCs) was recorded over time at 234 nm [58].

The AOA values of plant extracts were expressed in trolox equivalents (Tg_eq_). Calibration curves were constructed for 80% ethanol solutions of trolox (c = 1 m) injected in certain volumes into liposomes. For each concentration, measurements were carried out at least 3 times, and the measurement error did not exceed ±9%.

### 4.12. Statistical Analysis

All variants of the described experiments and assays were performed in triplicate. SigmaPlot 12.3 (http://www.sigmaplot.co.uk accessed on 9 February 2021) and Microsoft Excel were used for statistical processing. The tables and plots show arithmetic means (M) and their standard errors (±SEM). Superscripts show statistical significance of the differences between means according to Tukey’s test at *p* ≤ 0.05.

## 5. Conclusions

As a result of the conducted research, a comprehensive study of the morphophysiological characteristics, phenolic complex, and antioxidant activity of in vitro microshoots of three types of rhododendrons was carried out for the first time: evergreen *R. smirnowii*, semi-deciduous *R. PJM Elite*, and deciduous *R. japonicum*. According to the micropropagation protocol presented in this work, viable microshoots of rhododendrons were obtained with the preservation of species-specific features characteristic of intact plants. They differed in morphometric parameters (length, weight, branching pattern), the content of chlorophylls *a* and *b*, as well as the accumulation of PCs.

Using the HPLC method, a predominant content of flavonoids was shown in rhododendron microshoots. It is important to emphasize that in vitro microshoots retain the main species-specific features of the balance of the flavonoid nature metabolites characteristic of intact plants, although they have certain differences in their qualitative and quantitative composition. A high correlation was found between the AOA and the TPC, between the AOA and the flavonols content in the extracts, which confirms their key role in protecting cell membranes from oxidative damage.

The results obtained indicate the prospects of using rhododendron microshoots as an alternative source of plant bioantioxidants of phenolic nature. Of particular interest are the microshoots of *R. japonicum* with a high content of quercetin, kaempferol, and myricetin glycosides, as well as *R. PJM Elite* with an increased content of taxifolin and (-)-epicatechin, pharmacologically valuable compounds with pronounced antioxidant properties. All this indicates the prospects of using in vitro culture to obtain valuable biologically active substances from rhododendrons. Independence from seasonal and climatic factors, the possibility of standardizing growing conditions, and the high antioxidant activity of extracts make this technology attractive for the pharmaceutical and cosmetic industries.

## Figures and Tables

**Table 1:** Morphometric characteristics of microshoots of various *Rhododendron* species *.

Species	Shoot Fresh Weight, mg	Water Content, %	Shoot Length, mm	Number of Internodes	Branching Frequency, %
*R. smirnowii*	7.1 ± 0.5 ^b^	92.3 ± 0.2 ^b^	16.3 ± 1.1 ^c^	4.3 ± 0.1 ^b^	-
*R. PJM Elite*	13.1 ± 1.8 ^a^	93.1 ± 0.7 ^a^	31.1 ± 1.5 ^a^	9.2 ± 0.3 ^a^	3.1 ± 0.5 ^b^
*R. japonicum*	13.5 ± 1.9 ^a^	93.4 ± 0.5 ^a^	27.7 ± 1.2 ^b^	8.9 ± 0.5 ^a^	25.0 ± 0.7 ^a^

* The cultivation duration on the AM_3_ medium for elongation was 9–10 weeks. Results are expressed as means ± SD, *n* = 20. The significant differences at *p* < 0.05 are indicated by different Latin letters.

**Table 2:** The chlorophyll *a* and *b* content (mg/gRW) and their ratio in microshoots of various *Rhododendron* species.

Species	Chlorophyll			
	*a*	*b*	*a + b*	*a/b* Ratio
*R. smirnowii*	0.25 ± 0.011 ^b^	0.11 ± 0.004 ^b^	0.36 ± 0.018 ^b^	2.2 ± 0.098 ^a^
*R. PJM Elite*	0.35 ± 0.022 ^a^	0.19 ± 0.007 ^a^	0.54 ± 0.028 ^a^	1.8 ± 0.065 ^b^
*R. japonicum*	0.15 ± 0.006 ^c^	0.08 ± 0.003 ^c^	0.23 ± 0.012 ^c^	1.8 ± 0.061 ^b^

Results are expressed as means ± SD, *n* = 3. The significant differences at *p* < 0.05 are indicated by different Latin letters.

**Table 3:** Kinetic parameters of antiradical activity (ARA) for different rhododendron microshoot extracts.

Species	I, %	IC_50_, μg_dw_/mL	ARA_Tr_, μmolTr/mg_dw_
*R. smirnowii*	38.9 ± 1.7 ^c^	41.6 ± 3.7 ^a^	0.474 ± 0.024 ^c^
*R. PJM Elite*	44.5 ± 2.2 ^b^	37.3 ± 2.6 ^b^	0.548 ± 0.027 ^b^
*R. japonicum*	61.6 ± 3.1 ^a^	28.8 ± 1.8 ^c^	0.665 ± 0.039 ^a^

Parameters: I—inhibitory activity, %; IC_50_—the amount of extract required to scavenge 50% of DPPH radicals; ARA_Tr_—antiradical activity, expressed in Tr_eq_. Results are expressed as means ± SD, *n* = 3. The significant differences at *p* < 0.05 are indicated by different Latin letters.

**Table 4:** Kinetic parameters of antioxidant activity (AOA) for different rhododendron microshoot extracts.

Species	τ(C), y = a_n_x + b,	AOA_Tr_ ± SD, μmolTr/mg_dw_
*R. smirnowii*	y = 1933.3x + 4.6; R^2^ = 0.998	0.179 ± 0.011
*R*. *PJM Elite*	y = 1322.2x + 5.7; R^2^ = 0.993	0.155 ± 0.014
*R. japonicum*	y = 2160.6x + 4.8; R^2^ = 0.997	0.206 ± 0.018

y = a_n_x + b—regression equations of τ on C dependence; AOA_Tr_—AOA in eq.Tr.

## Data Availability

The data presented in this study are available upon request from the corresponding author.

## Supplementary Materials

The following supporting information can be downloaded at: https://www.mdpi.com/article/10.3390/molecules30142949/s1, Table S1: The phenolic compounds content in microshoots of various types of rhododendrons; Figure S1: HPLC-chromatograms of *R. smirnowii* (a), *R. PJM Elite* (b), *R. japonicum* (c) microshoot extracts at 270 nm (I) and 360 nm (II). On the *X*-axis: retention time, min; on the *Y*-axis: the detector signal, in units of optical density. 1—(−)-epicatechin, 2—syringic acid, 7—taxifolin, 12—hyperoside, 13—isoquercitrin, 16—myricetin, 17—avicularin, 18—quercitrin, 19—astragalin, 20—nicotiflorin, 23—quercetin, 28—kaempferol; Figure S2: Efficiency of DPPH radical reduction (%) at different concentrations of microshoot extracts of *R. smirnowii* (1), *R. PJM Elite* (2), *R. japonicum* (3). The exposure time is 30 min; Figure S3: Dependence of the induction period τ (τ-τ0) on the specific content of microshoot extracts of *R. smirnowii* (1), *R. PJM Elite* (2), *R. japonicum* (3) in PCh-liposomes.

## Abbreviations

The following abbreviations are used in this manuscript:

PCs	Phenolic compound
AOA	Antioxidant activity
BAS	Biologically active substances
AM	Anderson’s medium
ARA	Antiradical activity
DPPH	2,2-diphenyl-1-picrylhydrazyl
AO	Antioxidant
AAPH	2,2′-azobis-(amidinopropane)-dihydrochloride
PCh	Phosphatidylcholine
DC	Diene conjugates
