# Supplementary material for: Rhododendron Microshoot Culture as a Source of Phenolic Antioxidants for Biomedicine
Source: Molecules. 2025 Jul 12;30(14):2949. doi: 10.3390/molecules30142949 (PMC12300174; doi:10.3390/molecules30142949)
Supplement: Supplementary file 1 [file molecules-30-02949-s001.zip › molecules-3722590-supplementary.pdf]

Table S1. The phenolic compounds content in microshoots of various types of rhododendrons.

Peak №	Compound	Spectral characteristics, λmax, nm	Retention time (tR), min	Content, mg/gDW		
				R. smirnowii	*R. PJM Elite*	*R. japonicum*
1	(-)-epicatechin	235, 280	3.6	0.32	2.42	1.59
2	Syringic acid	275	4.2	0.36	0.40	0.18
3	PC	-	4.7	0	0.32	0.15
4	PC	210, 225sh, 280, 325sh	5.3	0.50	1.66	0
5	PC	-	6.2	0.22	0.22	0.18
6	PC	225, 290	6.8	1.51	0	1.33
7	Taxifolin	290	7.5	0.31	1.26	0.51
8	PC	255, 360	9,9	0	0	0
9	PC	270	10,5	0	0	0
10	PC	265, 360	11.7	0	0	0.20
11	PC	260, 360	14,1	0	0	0
12	Hyperoside	255, 355	17.2	0.58	0.31	0.49
13	Isoquercitrin	260, 265sh, 360	18.3	0.39	0.41	0.44
14	Rutin	256, 358	19.1	0	0	0
15	PC	255, 310, 360	22.4	0	0	0.27
16	Myricetin	255, 300sh, 375	25.2	0	0	0
17	Avicularin	260, 270sh, 360	27.0	0	0	0.81
18	Quercitrin	256, 350	29.8	1.71	0.45	3.18
19	Astragalin	-	31.6	0.38	0	0.79
20	Nocotiflorine	260, 290sh, 350	33.7	0.25	0	1.01
21	PC	265, 350	35.6	0.50	0	0.45
22	PC	265, 355	39.9	0.15	0	0.25
23	Quercetin	255, 372	40.6	0.17	0.10	0.24
24	PC	250, 310sh, 350	41.2	0.17	0.09	0.21
25	PC	-	42.0	0.34	0	0
26	PC	260, 360	43.9	0.35	0	0.20
27	PC	265, 355	44.8	0.62	0.11	0.27
28	Kaempferol	-	46.5	0.14	0	0
29	PC	-	48.6	0.17	0	0
**Total PCs**				**9.14**	**7.75**	**12.75**
Including glycosides of						
Quercetin				2.74	2.19	4.9
Kaempferol				1.02	0	2.09
Myricetin				0	0.47	0.65
**Total content of flavonols**				**3.76**	**2.57**	**7.64**

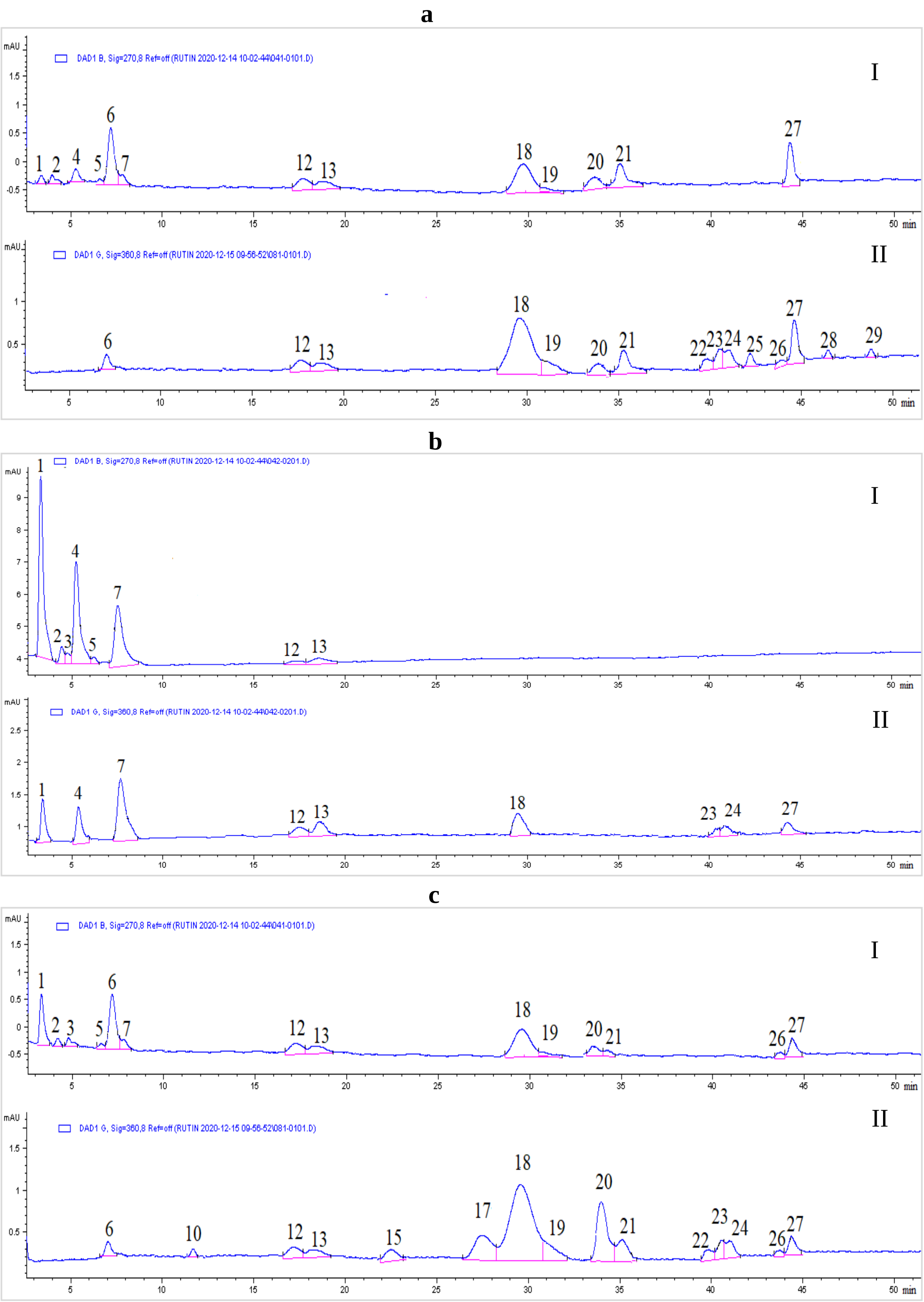

Figure S1. HPLC-chromatograms of *R. smirnowii* (**a**), *R. PJM Elite* (**b**), *R. japonicum* (**c**) microshoot extracts at 270 nm (**I**) and 360 nm (**II**). On the X-axis: retention time, min; on the Y-axis: the detector signal, in units of optical density.

1 – (-)-epicatechin, **2** - syringic acid, **7** - taxifolin, **12** – hyperoside, **13** – Isoquercitrin, **16** – myricetin, **17** – avicularin, **18** - quercitrin, **19** – astragalin, **20** – nicotiflorin, **23** – quercetin, **28** – kaempferol

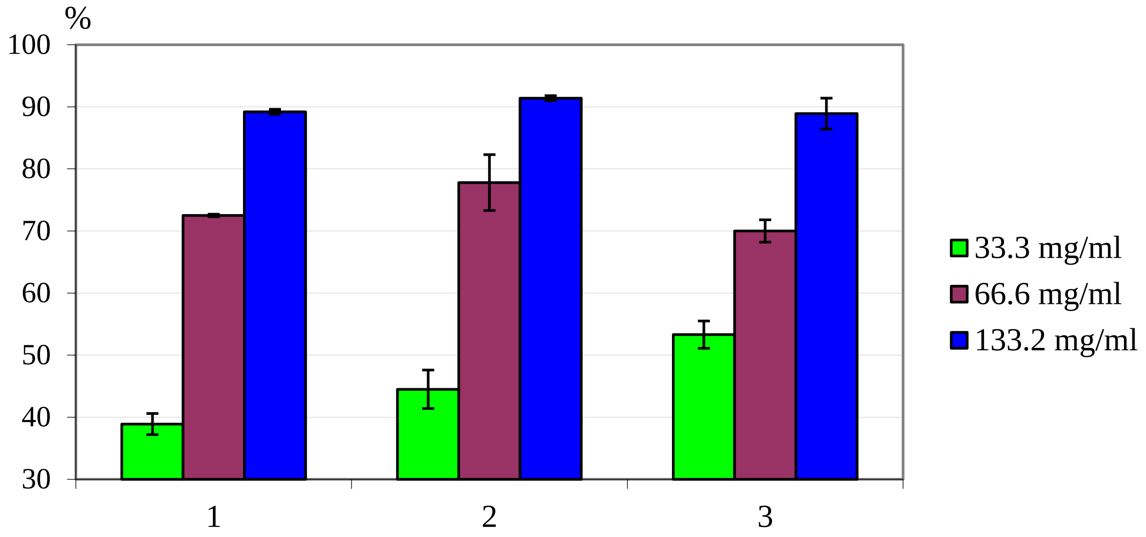

Figure S2. Efficiency of DPPH radical reduction (%) at different concentrations of microshoots extracts of *R. smirnowii* (1), *R. PJM Elite* (2), *R. japonicum* (3). The exposure time is 30 minutes.

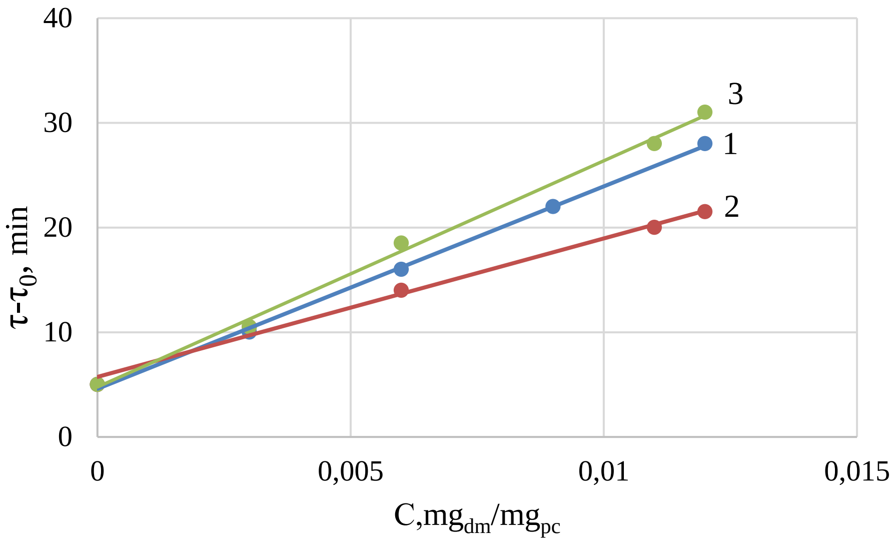

Figure S3. Dependence of the induction period τ (τ-τ0) on the specific content of microshoots extracts of *R. smirnowii* (1), *R. PJM Elite* (2), *R. japonicum* (3) in PCh-liposomes.
